# Supplementary material for: Genome-Wide Identification of the B-Box Gene Family and Expression Analysis Suggests Their Potential Role in Photoperiod-Mediated β-Carotene Accumulation in the Endocarp of Cucumber (Cucumis sativus L.) Fruit
Source: Genes (Basel). 2022 Apr 8;13(4):658. doi: 10.3390/genes13040658 (PMC9031713; doi:10.3390/genes13040658)
Supplement: Supplementary file 1 [file genes-13-00658-s001.zip › 6. Additional File 1_Table S1_S4.pdf]

Table S1. The sequence analysis and weblog of 20 identified motifs of the *CsaBBX* genes family in cucumber

Motifs	E-value	Sites	Width	Amino acid sequences	Logo
1	5.8e-396	26	31	VYCKADEAALCLSCDA KVHSANKLARRHQRV	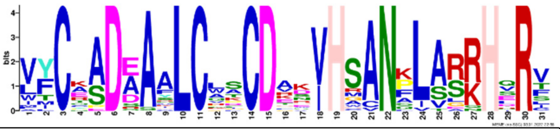
2	2.6e-310	12	50	QJSGMDREARVLRYRE KRKTRKFEKTIRYASR KAYAETRPRVKGRFVK RT	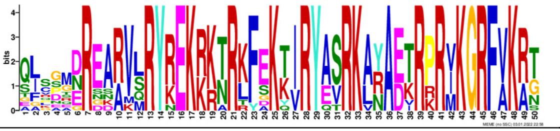
3	1.2e-153	17	30	LCDICKSAPAFLFCRE DRAFLCLECDAKIH	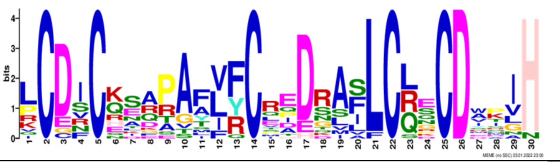
4	6.8e-045	26	11	ILCDVCEQARA	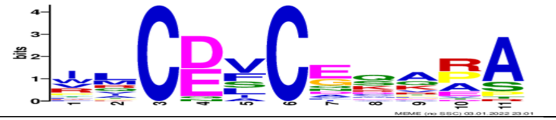
5	1.5e-026	4	41	RSKLNSFGYQPQSLSH SVSSSSLDVGVVPDG NSMSDVSYPM	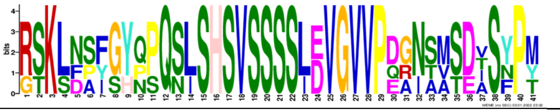
6	1.7e-016	5	26	HKRQPINGYSGCPSAA ELSSJWSFDL	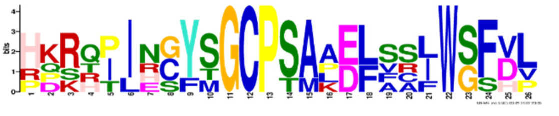
7	2.9e-015	7	16	EHTRKHKRFLLTGVKV	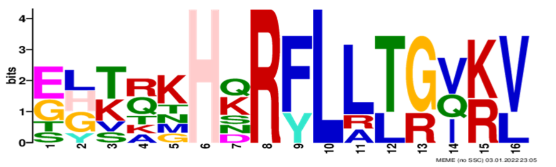
8	2.9e-015	3	50	KIKNKKKIISLRLDYEA VIAAWGSQGSPWTSG BRPBFDLDDYWPDWM GVC	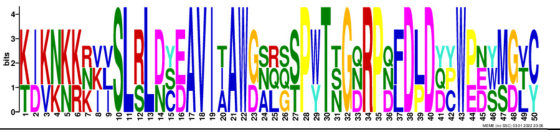
9	6.2e-014	4	18	YLLETLPGWHFEEFLD SS	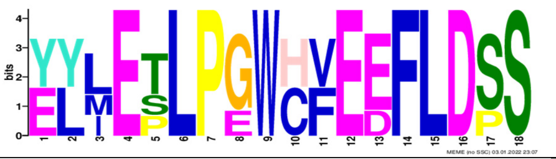
10	1.6e-009	6	16	EEVEAASWLLMNPPPN	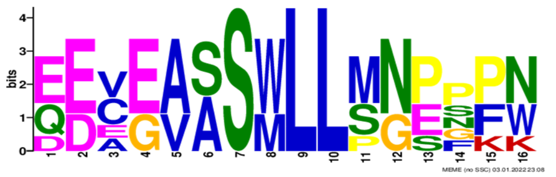

11	1.3e-009	2	36	HNDIKDSIVPVQTKPD PTPVINQTHSPENCYD IEFC	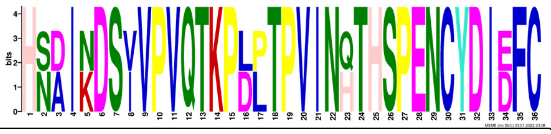
12	1.2e-008	3	17	HLFFSEMDSFIDFEYP N	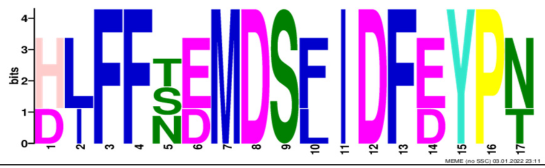
13	7.4e-008	3	29	MLSEVDEIYGSAASGV LLTDAQYGVVPTF	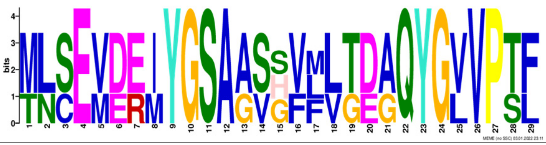
14	3.7e-007	4	16	LLCNVCQSPTPWYLSG	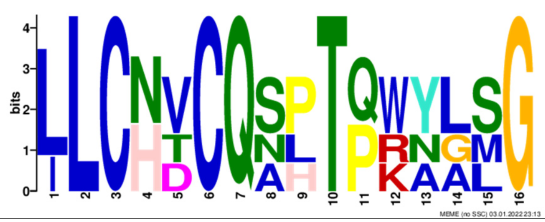
15	2.9e-006	2	48	MQPANSNGASADSVM SAKTEPILCFNNKQEQ SGLSFSGLTGESSAGE H	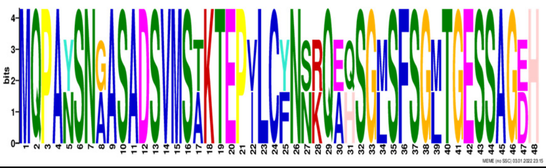

Table S2. Duplicated *CsaBBX* genes in cucumber and the dates of their duplications

Gene_1	Gene_2	Ka	Ks	Ka_Ks	Duplication Type	T(MYA)	Note
CsaBBX1	*CsaBBX18*	0.422938	na	na		-	High Sequence Divergence Value (pS>=0.75)
CsaBBX11	*CsaBBX22*	0.432162	na	na	Segmental	-	High Sequence Divergence Value (pS>=0.75)
CsaBBX20	*CsaBBX24*	0.241592	1.415665	0.170656	Segmental	107.9013	
CsaBBX13	*CsaBBX21*	1.609262	1.423268	1.130681	Segmental	108.4808	
CsaBBX6	*CsaBBX23*	0.304801	1.716601	0.177561	Segmental	130.8385	
CsaBBX3	*CsaBBX4*	0.074849	0.366775	0.204072	Tandem	27.95541	
CsaBBX10	*CsaBBX25*	0.377614	na	na	Segmental	-	High Sequence Divergence Value (pS>=0.75)

T= KS/2λ; where $\lambda = 6.56\times10^{-9}$

Table S3: Syntenic genes between cucumber *CsaBBX* genes and Arabidopsis *AthBBXs* genes

Arabidopsis				Cucumber		
**Chr**	**Gene ID**	**BBX**		**Chr.**	**Gene ID**	**Gene name**
1	transcript:AT1G73870.1	*AthBBX16*	==	Chr1	Csa1G023050.1	*CsaBBX1*
1	transcript:AT1G68520.1	*AthBBX14*	==	Chr1	Csa1G023050.1	*CsaBBX1*
1	transcript:AT1G75540.1	*AthBBX21*	==	Chr2	Csa2G365080.1	*CsaBBX7*
1	transcript:AT1G75540.1	*AthBBX21*	==	Chr2	Csa2G250430.1	*CsaBBX6*
1	transcript:AT1G78600.2	*AthBBX22*	==	Chr4	Csa4G083550.1	*CsaBBX14*
1	transcript:AT1G25440.1	*AthBBX15*	==	Chr5	Csa5G609670.1	*CsaBBX16*
1	transcript:AT1G68190.2	*AthBBX27*	==	Chr5	Csa5G610520.1	*CsaBBX17*
1	transcript:AT1G68520.1	*AthBBX14*	==	Chr5	Csa5G609670.1	*CsaBBX16*
1	transcript:AT1G68520.1	*AthBBX14*	==	Chr6	Csa6G039540.1	*CsaBBX18*
1	transcript:AT1G75540.1	*AthBBX21*	==	Chr7	Csa7G004690.1	*CsaBBX23*
2	transcript:AT2G24790.1	*AthBBX4*	==	Chr2	Csa2G383330.1	*CsaBBX8*
3	transcript:AT3G21880.2	*AthBBX10*	==	Chr2	Csa2G423550.1	*CsaBBX9*
3	transcript:AT3G21890.1	*AthBBX31*	==	Chr2	Csa2G423560.1	*CsaBBX10*
3	transcript:AT3G07650.4	*AthBBX7*	==	Chr6	Csa6G113560.1	*CsaBBX20*
3	transcript:AT3G07650.4	*AthBBX7*	==	Chr7	Csa7G031530.1	*CsaBBX24*
3	transcript:AT3G21890.1	*AthBBX31*	==	Chr7	Csa7G044810.1	*CsaBBX25*
4	transcript:AT4G39070.1	*AthBBX20*	==	Chr2	Csa2G250430.1	*CsaBBX6*
4	transcript:AT4G39070.1	*AthBBX20*	==	Chr2	Csa2G365080.1	*CsaBBX7*
4	transcript:AT4G27310.1	*AthBBX28*	==	Chr3	Csa3G850640.1	*CsaBBX11*
4	transcript:AT4G38960.3	*AthBBX19*	==	Chr6	Csa6G505230.1	*CsaBBX21*
4	transcript:AT4G27310.1	*AthBBX28*	==	Chr7	Csa7G044810.1	*CsaBBX25*
4	transcript:AT4G39070.1	*AthBBX20*	==	Chr7	Csa7G004690.1	*CsaBBX23*
5	transcript:AT5G57660.1	*AthBBX6*	==	Chr1	Csa1G420310.1	*CsaBBX3*
5	transcript:AT5G57660.1	*AthBBX6*	==	Chr2	Csa2G057080.1	*CsaBBX5*
5	transcript:AT5G24930.1	*AthBBX5*	==	Chr2	Csa2G383330.1	*CsaBBX8*
5	transcript:AT5G48250.1	*AthBBX8*	==	Chr6	Csa6G113560.1	*CsaBBX20*
5	transcript:AT5G48250.1	*AthBBX8*	==	Chr7	Csa7G031530.1	*CsaBBX24*

Table S4: Primer sequences used for qRT-PCR analysis.

Gene Name	Gene ID	Accession number	Forward primer (5'-3')	Reverse primer (5'-3')	Sequence length (bp)	Product size (bp)
CsaBBX4	*Csa1G420320*	KGN65418	TTGTGAAGGGTTTCGGCG	CCGAAACCCTTCACAACA	1128	191
CsaBBX5	*Csa2G057080*	KGN61122	GCGTTCCCGCCTCTACCAAA	TTTGGTAGAGGCGGGAAC	960	183
CsaBBX9	*Csa2G423550*	KGN63275	CCTCTATGCGAGTTCTGTG	TCCACAGAACTCGCATAGA	1191	120
CsaBBX11	*Csa3G850640*	KGN59860	CTAACCTCTGTTGGGACTGC	CATCGCAGTCCCAACAGA	825	186
CsaBBX13	*Csa4G047370*	KGN53325	GCTGCTGCTATTGTCTTT	GCCTTTTCACAAGCGTCA	561	115
CsaBBX15	*Csa4G124910*	KGN53769	ATGTTGAAGACAGAAGACGAG	CTGCTGGACATCTCTCACA	1035	142
CsaBBX17	*Csa5G610520*	KGN52118	CTTTGCCTTCCCTGTGATG	CATCACAGGGAAGGCAAAG	1464	117
CsaBBX22	*Csa6G516780*	KGN49170	ATGAAGAAGTTGTGCGAGC	GCTCGCACAACTTCTTCAT	372	125
CsaBBX23	*Csa7G004690*	KGN43167	GCCTCTGTTTTTTGTTATGC	GCATAACAAAAAACAGAGGC	669	129
CsaBBX25	*Csa7G044810*	KGN43541	AGGTGTAGAACAGGAAGAAACG	GGGTCGTTTCTTCCTGTTCTA	399	109
Csaβ-Actin	*Csa2G301530*	KGN62135	ATTGTTCTCAGTGGTGGTTCTAC	CCTTTGAGATCCACATCTGCT	1134	190
