# Supplementary material for: Genome-Wide Identification of the B-Box Gene Family and Expression Analysis Suggests Their Potential Role in Photoperiod-Mediated β-Carotene Accumulation in the Endocarp of Cucumber (Cucumis sativus L.) Fruit
Source: Genes (Basel). 2022 Apr 8;13(4):658. doi: 10.3390/genes13040658 (PMC9031713; doi:10.3390/genes13040658)
Supplement: Supplementary file 1 [file genes-13-00658-s001.zip › 7. Addition File 2_Figure S1_S4.pdf]

Supplementary Figures

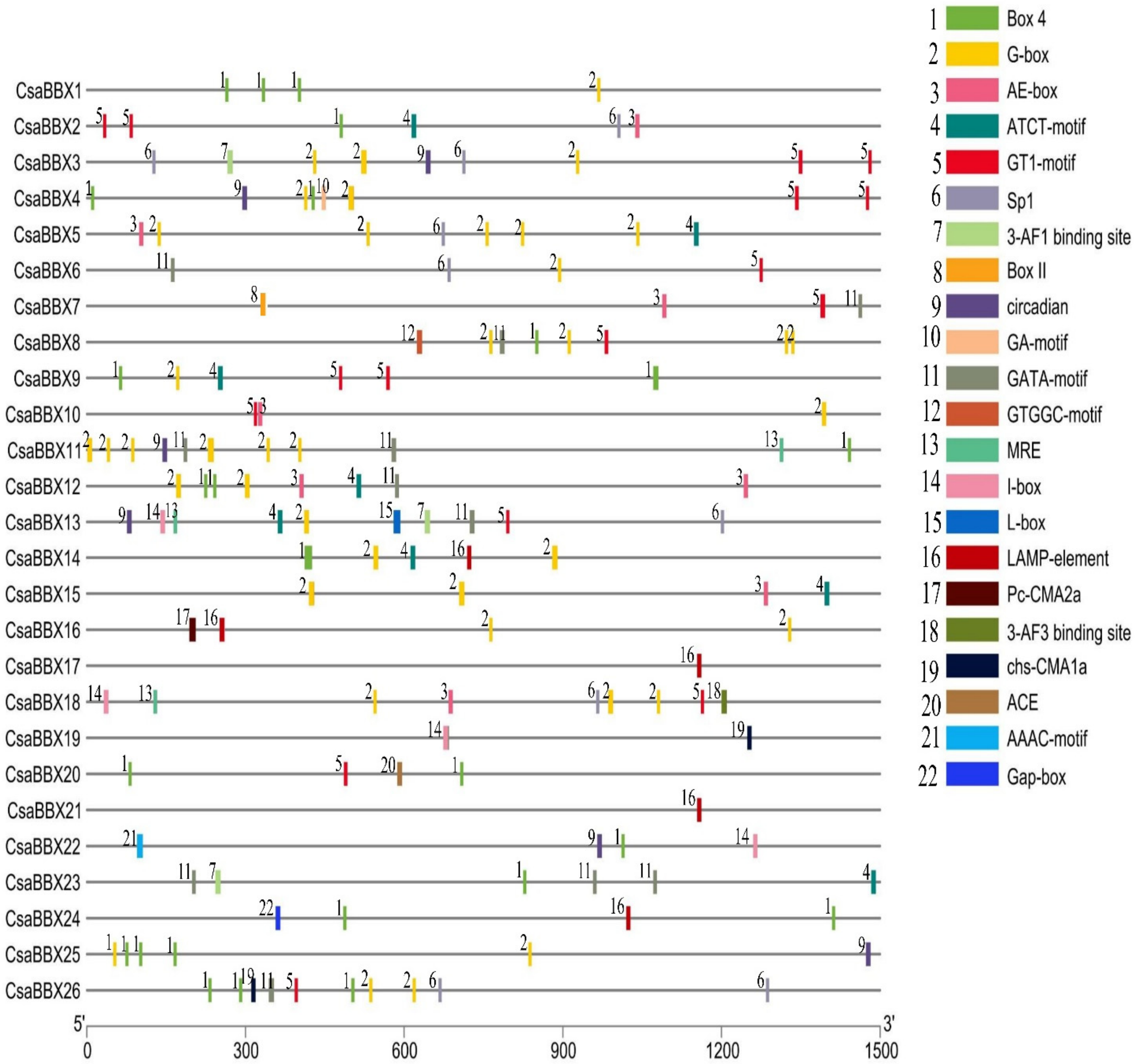

Figure S1. Promoter cis-element analysis of the light-responsive motif of *CsaBBX* in cucumber. The distribution of the light-responsive cis-acting elements in the 1.5kb upstream promoter region of *CsaBBX* genes was detected via the Plant CARE website. Different colors marked by numbers represent different types of cis-elements

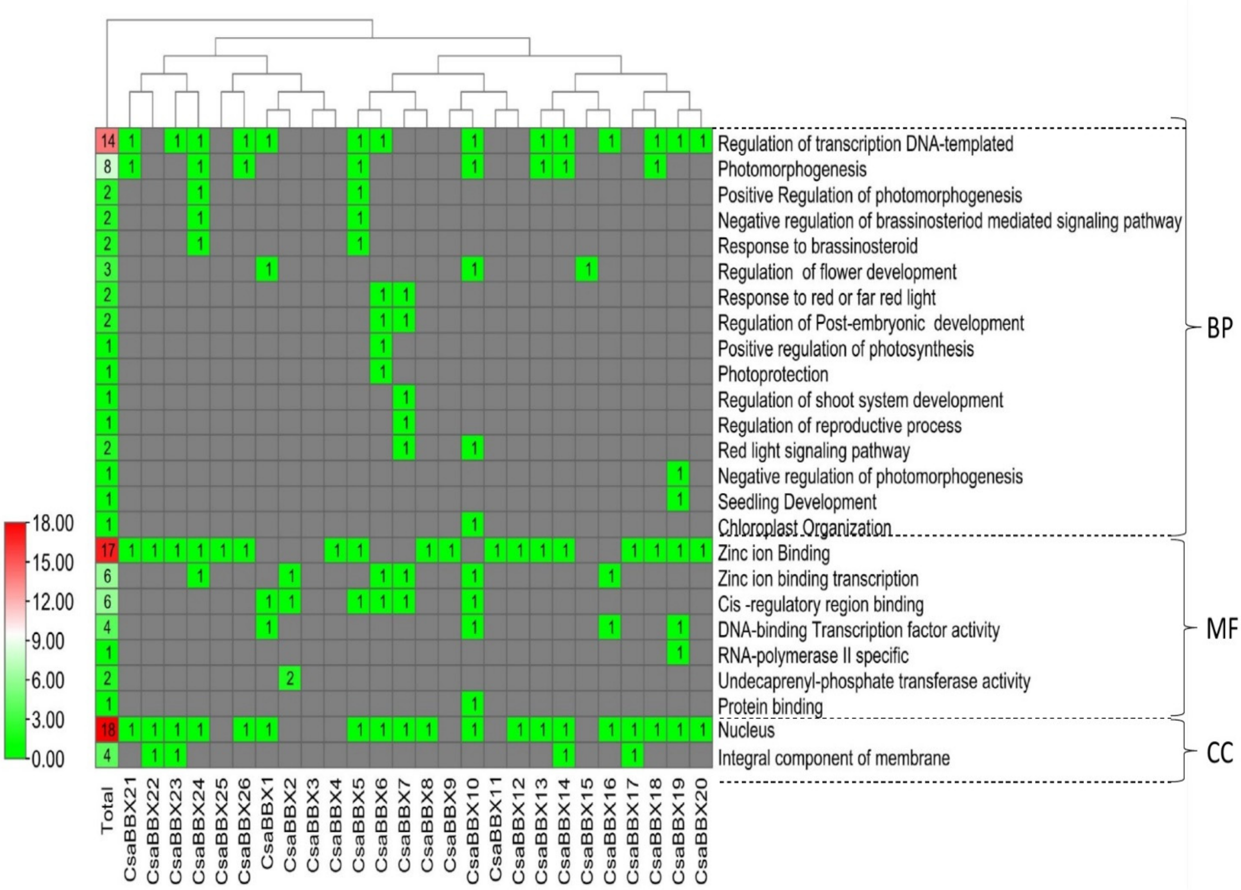

Figure S2. Distribution of gene ontology enrichment among *CsaBBX* genes. The heatmap was generated through TBTOOLs. Numbers in boxes are the number of genes per GO component. The column labeled "Total" refers to the total number of *CsaBBX* in each category. BP-Biological process, MF- Molecular function, CC- cellular component.

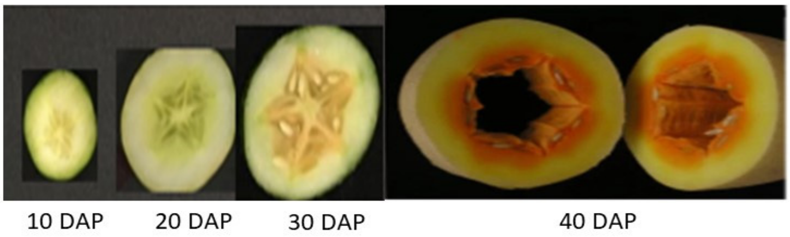

Figure S3. Endocarp morphological feature of Orange-fleshed cucumber. Endocarp flesh color changes at four developmental time points of fruits at 10, 20, 30 and 40 days after pollination (DAP).

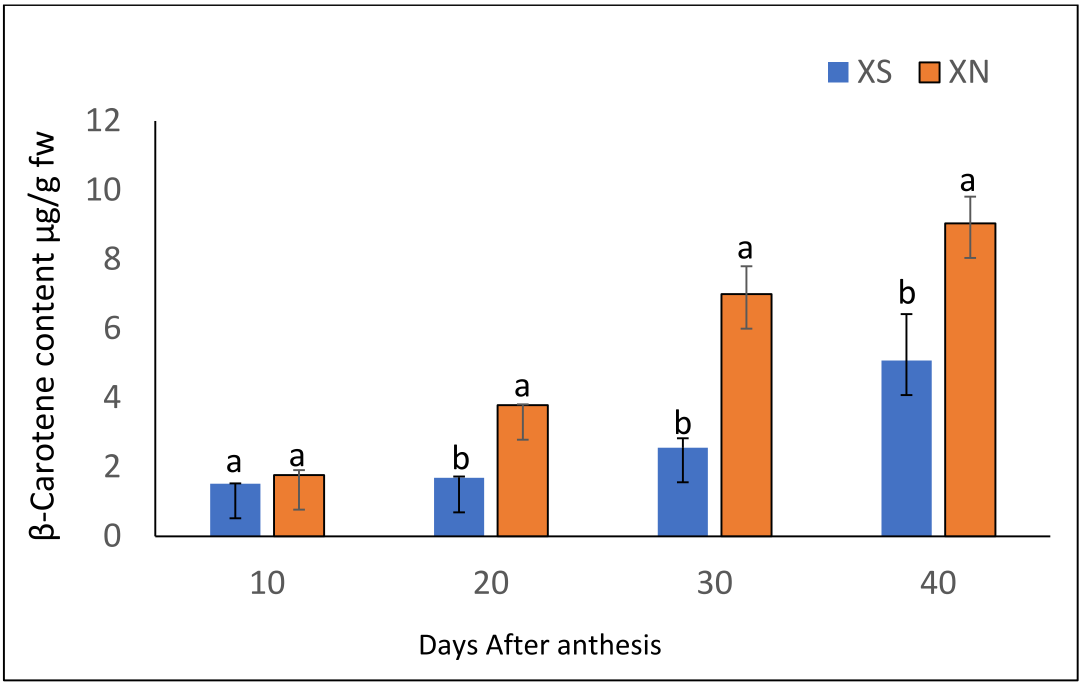

Figure S4. Changes in β-carotene levels (µg/g fresh weight; fw) in the endocarp during fruit development under short (S) and neutral (N) day conditions. Bars with the different letters on each sampling date are significantly different while with the same letters are not significantly different according to the t-test ($p \leq 0.05$). Error bars indicate the standard deviation ($n = 3$).
